# Impact of *Coxiella burnetii* vaccination on humoral immune response, vaginal shedding, and lamb mortality in naturally pre-infected sheep

**DOI:** 10.3389/fvets.2022.1064763

**Published:** 2022-12-19

**Authors:** Benjamin Ulrich Bauer, Clara Schoneberg, Thea Louise Herms, Sven Kleinschmidt, Martin Runge, Martin Ganter

**Affiliations:** ^1^Clinic for Swine and Small Ruminants, Forensic Medicine and Ambulatory Service, University of Veterinary Medicine Hannover, Foundation, Hanover, Germany; ^2^Department of Biometry, Epidemiology and Information Processing, WHO Collaborating Centre for Research and Training Health at the Human-Animal-Environment Interface, University of Veterinary Medicine Hannover, Foundation, Hanover, Germany; ^3^Food and Veterinary Institute Braunschweig/Hannover, Lower Saxony State Office for Consumer Protection and Food Safety (LAVES), Hanover, Germany

**Keywords:** abortion, *Coxiella burnetii*, IgG, lamb losses, Q fever, sheep, vaccination, zoonosis

## Abstract

**Introduction:**

Sheep are considered to be one of the main reservoirs for *Coxiella burnetii*, a gram-negative bacterium with high zoonotic potential. Infected sheep shed tremendous amounts of the pathogen through birth products which caused human Q fever epidemics in several countries. Information about the impact of an inactivated *C. burnetii* Phase I vaccine on humoral immune response, vaginal shedding, and lamb mortality in naturally pre-infected sheep is scarce.

**Methods:**

Two identically managed and naturally *C. burnetii*-infected sheep flocks were examined for two lambing seasons (2019 and 2020). One flock (VAC) received a primary vaccination against Q fever before mating and the second flock served as control (CTR). In each flock, one cohort of 100 ewes was included in follow-up investigations. Serum samples at eight different sampling dates were analyzed by *C. burnetii* phase-specific ELISAs to differentiate between the IgG Phase I and II responses. Vaginal swabs were collected within three days after parturition and examined by a *C. burnetii* real-time PCR (IS*1111*). Lamb losses were recorded to calculate lamb mortality parameters.

**Results:**

After primary vaccination, almost all animals from cohort VAC showed a high IgG Phase I response up until the end of the study period. In cohort CTR, the seropositivity rate varied from 35.1% to 66.3%, and the Phase I and Phase II pattern showed an undulating trend with higher IgG Phase II activity during both lambing seasons. The number of vaginal shedders was significantly reduced in cohort VAC compared to cohort CTR during the lambing season in 2019 (*p* < 0.0167). There was no significant difference of vaginal shedders in 2020. The total lamb losses were low in both cohorts during the two investigated lambing seasons (VAC 2019: 6.8%, 2020: 3.2%; CTR 2019: 1.4%, 2020: 2.7%).

**Discussion:**

Neither the *C. burnetii* vaccine nor the *C. burnetii* infection seem to have an impact on lamb mortality. Taken together, the inactivated *C. burnetii* Phase I vaccine induced a strong IgG Phase I antibody response in naturally pre-infected sheep. It might also reduce vaginal shedding in the short term but seems to have little beneficial impact on lamb mortality.

## Introduction

*Coxiella burnetii* is a gram-negative bacterium with high zoonotic potential, with ruminants being the main reservoir. Inhalation of contaminated aerosols and dust lead to infection with *C. burnetii* in humans and animals, respectively. Most human Q fever epidemics are associated with aborting or lambing sheep and goats (1, 2). Tremendous amounts of the pathogen are shed through birth products. Acute clinical symptoms in humans are shown by approximately half of the infected people mostly having severe flu-like symptoms (3). As a consequence of an acute infection, Q fever fatigue syndrome can occur in up to 20% of the patients and can last for several years (4). A small number of infected individuals develop chronic Q fever, which manifests as endocarditis or infections of aneurysms or vascular prostheses (5).

Abortion, stillbirth, and postpartum diseases such as retained placenta and (endo)metritis have been associated with *C. burnetii* infection in dairy cattle, but definitive evidence that *C. burnetii* is the causative agent is lacking (6, 7). In goat flocks, *C. burnetii* causes endemic abortion with losses of up to 90% or weak kids with low body weight (8, 9). Metritis can be present in goats after *C. burnetii* abortion (9), but this was not confirmed under experimental conditions (8). Experimentally infected sheep did not abort but gave birth to non-viable lambs (10). Under field conditions, *C. burnetii* shedding ewes gave birth to healthy lambs (11–14) and the abortion rate in infected sheep flocks might be <3% (15–18). Such a low number is tolerated in healthy sheep flocks and investigations are usually not initiated (19, 20). Co-infections with other abortifacient agents like *Chlamydia (Chl.) abortus, Toxoplasma (T.) gondii*, and border disease virus increases the abortion rates in a *C. burnetii*-infected sheep flock (21–25) and leads to a potential adverse overestimation of *C. burnetii* on the reproductive performance. Different ovine abortifacient agents such as *Chl. abortus* and *Listeria monocytogenes*, cause brownish vaginal exudate, placental retention, and endometritis (26, 27), but the impact of *C. burnetii* on the ewes' health post-partum is still unknown. Moreover, data about the effect of an inactivated *C. burnetii* Phase I vaccine on perinatal complications in sheep are missing.

*C. burnetii* undergoes a lipopolysaccharide (LPS) phase variation in which its virulent smooth LPS phase, Phase I (PhI), converts to an avirulent rough LPS phase, Phase II (PhII), upon a serial passage in non-immunocompetent subjects (28, 29). Both phase variations are used for diagnostic purposes to determine the clinical status of a *C. burnetii* infection. In general, an increase in IgG PhII antibodies without or with a low IgG PhI response is interpreted as a recently acquired infection (30, 31). The presence of IgG PhI alone or in combination with similar or lower IgG PhII antibody levels has been associated with antigen contact in the past (32, 33). Recently, the phase-specific serology was used to characterize the humoral immune response in infected *C. burnetii* sheep flocks after vaccination (33, 34). Due to the long-lasting IgG PhI response induced by the vaccine, booster vaccination for *C. burnetii*-infected and vaccinated sheep does not appear to be necessary, but further studies are needed to prove this concept (33).

In the mouse model, antibodies were unable to completely control *C. burnetii* infection but prevented the development of clinical disease at an early stage (35, 36). Moreover, the antibody response alone was unable to eliminate a *C. burnetii* infection (37), but B cells might play a crucial role in clearance of *C. burnetii* and in regulation of inflammatory response (38). The TH1-mediated cellular immunity was based on the release of proinflammatory cytokines such as interferon-γ (INF-γ). Impaired or absent INF-γ production by T cells resulted in an increased mortality rate of infected mice (39). Consequently, protective immunity is only achieved by humoral and cell-mediated immunity. The cell-mediated immunity eliminates the pathogen, and specific antibodies accelerate this process (40). To date, routine diagnostic assays for evaluating the cell-mediate response of *C. burnetii* are not available for large sample quantities in veterinary medicine.

Since 2010, a formalin-inactivated *C. burnetii* Nine Mile strain Phase I whole-cell vaccine (Coxevac^®^, CEVA Santé Animale, Libourne, France) is licensed for cattle and goats in several European countries (41). This vaccine is also commonly applied to sheep with 1 mL per vaccine dose, but information about efficiency is scarce (33). A decrease in vaginal shedding after vaccination was reported in several studies (16, 23, 33), but a significant difference was not achieved between non-vaccinated and immunized sheep (42, 43). Furthermore, vaginal shedding was also reduced in some flocks at the following lambing season without vaccination, indicating a self-limitation of Q fever in sheep flocks (15, 44, 45). Assumably, an increasing herd immunity contributes to the interruption of infection cycle, but this hypothesis needs further investigations (34, 42).

The present field study had two major objectives. Firstly, to evaluate the effect of an inactivated *C. burnetii* Phase I vaccine on phase-specific IgG response and vaginal pathogen shedding in a pre-infected flock. Secondly, to investigate the influence of an inactivated *C. burnetii* Phase I vaccine on perinatal complications in ewes and lambs. For both these aims, two identically managed naturally pre-infected *C. burnetii* sheep flocks were examined during two subsequent lambing seasons. One sheep flock was vaccinated against *C. burnetii*, and the second flock served as control.

## Materials and methods

### Flock description and Q fever history

The extensive sheep farm consisted of two sheep flocks (CTR and VAC) which were kept strictly separated because they were located in two different German federal states, namely Schleswig-Holstein and Mecklenburg-Western Pomerania. Each flock contained approximately 800 crossbred ewes (German Blackheaded Mutton x Coburg Fox). Both extensive flocks were managed identically by the same farmer. In addition, flock VAC was taken care of by shepherd A and flock CTR by shepherd B. Each year, the two flocks lambed consecutively on pasture in spring and ewes with lambs were transported to the same lambing barn and stayed there for less than five days. Afterwards, ewes and lambs were returned to the pastures, but animals from each flock were never mixed with the other flock.

In April 2017, flock CTR suffered several abortions and *C. burnetii* was diagnosed in one placenta from an aborted sheep (Cq 31; LSI VetMAX^TM^*Coxiella burnetii*, Thermo Fisher Scientific, Germany). Moreover, a proportion of 11.8% of examined ewes (*n* = 17) had antibodies against *C. burnetii* (Q Fever Antibody Test Kit, IDEXX, Liebefeld, Switzerland). At the same time, preputial swabs from 12 breeding sires were collected as a novel diagnostic tool to detect *C. burnetii* DNA (46), and five of these samples tested *C. burnetii* positive (Cq 37, 38, 38, 40, 42). In December 2017, blood samples were collected from 90 sheep for the annual flock health check. These specimens were also analyzed for *C. burnetii* antibodies by an ELISA (Q Fever Antibody Test Kit, IDEXX, Liebefeld, Switzerland), and a detection rate of 6.7% was obtained. In April 2018, flock CTR was revisited, and vaginal swabs and blood samples from 42 ewes were collected after lambing. None of the vaginal swabs tested *C. burnetii* DNA positive by real-time PCR (LSI VetMAX^TM^*Coxiella burnetii*, Thermo Fisher Scientific, Germany), but 16.7% of the animals showed a *C. burnetii* antibody response analyzed by the commercial ELISA (Q Fever Antibody Test Kit, IDEXX, Liebefeld, Switzerland).

In flock VAC, no reproductive problems occurred during the lambing season from March to April 2017. Nevertheless, samples were also collected from flock VAC after lambing in April 2017 to determine a possible Q fever infection. *C. burnetii* antibodies were detected in one female sheep (*n* = 19; Q Fever Antibody Test Kit, IDEXX, Liebefeld, Switzerland), and one preputial swab from a breeding ram (*n* = 11) tested *C. burnetii* positive by real-time PCR (Cq 35; LSI VetMAX^TM^*Coxiella burnetii*, Thermo Fisher Scientific, Germany). In December 2017, a low intra-flock seroprevalence of 2.3% was determined during a Q fever study by a commercial ELISA, and all preputial (*n* = 21) and pre-lambing vaginal swabs (*n* = 20) tested negative (46). However, 35 vaginal swabs (*n* = 44) tested *C. burnetii* positive (Cq 22–40; LSI VetMAX^TM^*Coxiella burnetii*, Thermo Fisher Scientific, Germany) taken from the lambing sheep in April 2018, and 15.9% of these animals had a positive *C. burnetii* antibody activity (Q Fever Antibody Test Kit, IDEXX, Liebefeld, Switzerland).

An overview of the Q fever history of both flocks from April 2017 to April 2018 is given in Table 1.

**Table 1:** Q fever history of two identically managed and naturally infected sheep flocks.

**Flock**	**April 2017**	**October 2017**	**December 2017**	**April 2018**
CTR	• Abortion material: *C. burnetii* DNA (Cq 31) • 11.8% *C. burnetii* antibody positive ewes (*n* = 17) • Five *C. burnetii* DNA positive rams (preputial swabs, *n* = 12; Cq 37, 38, 38, 40, 42)	6.7% *C. burnetii* antibody positive sheep (*n* = 90)	Not applicable	• 16.7% *C. burnetii* antibody positive ewes (*n* = 42) • Vaginal swabs *C. burnetii* DNA negative (*n* = 42)
VAC	• No reproductive issues, 5.3% *C. burnetii* antibody positive ewes (*n* = 19) • One *C. burnetii* DNA positive ram (preputial swabs, *n* = 11; Cq 35)	Not applicable	• 2.3% *C. burnetii* antibody positive sheep (*n* = 44) • Preputial swabs (*n* = 24) and pre-lambing vaginal swabs (*n* = 20) *C. burnetii* DNA negative	• 15.9% *C. burnetii* antibody positive ewes (*n* = 44) • 35 *C. burnetii* DNA positive ewes (post-lambing vaginal swabs; *n* = 44; Cq 22-40)

Cq, cycle quantification.

In July 2020, both shepherds (A and B) were tested for *C. burnetii* antibodies by a semi-quantitative Q fever immunofluorescence assay, IgG, (Focus Diagnostics, Cypress, CA, USA), as described elsewhere (47) per the treating physician's request. The following IgM and IgG titers were detected in shepherd A: IgM Ph I and Ph II negative, IgG Ph I and Ph II: 1:64; shepherd B: IgM Ph I 1:32, IgM Ph II <1:16, IgG Ph I 1:64, IgG Ph II: 1:4,096.

### Vaccination

We decided to vaccinate all ewes (excluding gimmers) from flock VAC due to the detection of *C. burnetii* DNA during the lambing season in 2018 and to leave flock CTR unvaccinated as a control. This decision was supported by the assumption that the zoonotic risk from flock CTR was possibly lower due to the missing pathogen detection in sheep examined in April 2018. All ewes from flock VAC received 1 mL of an inactivated *C. burnetii* Phase I vaccine (Coxevac^®^, Ceva, Libourne, France, ChB: 1010FG1B) in August 2018. The volume of 1 mL contains 72 Q fever units (relative potency of Phase I antigen measured by ELISA in comparison with a reference item) and is approximately equivalent to 100 mg of inactivated corpuscular Phase I antigen of *C. burnetii* according to the manufacturer. A skin fold behind the shoulder was created to inject the vaccine subcutaneously with an automatic syringe (HSW ECO-MATIC, 1 mL, Henke Sass Wolf GmbH, Tuttlingen, Germany). Injection was performed with a new needle (Hypodermic-Needle, 20Gx1½”, WDT, Garbsen, Germany) for every 10th sheep. Three weeks later, the second dose was given, and thereby the primary vaccination was completed 4 weeks before tupping as recommended by the manufacturer. Flock VAC was only vaccinated in 2018, and no booster vaccination was administered in 2019.

### Study cohorts

The number of ewes required from each flock to estimate the vaccine impact on vaginal shedding was calculated on the assumption of 2% and 20% expected vaginal shedders in the vaccinated and control flock, respectively, 90% power and 5% precision. Therefore, 100 healthy multiparous ewes aged between three and four years from each flock were included in the investigations. These animals were clinically examined and individually ear-tagged (Twintag, Kleißner, Tauberbischofsheim, Germany). Thereby, an individual follow-up of every study ewe was possible during the entire study period from August 2018 to July 2020. The amount of study ewes declined in both flocks due to animal losses in summer/winter 2019, and a reduced number of ewes were available during the lambing season in 2020.

### Blood sample and vaginal swab collection

**Figure 1:**
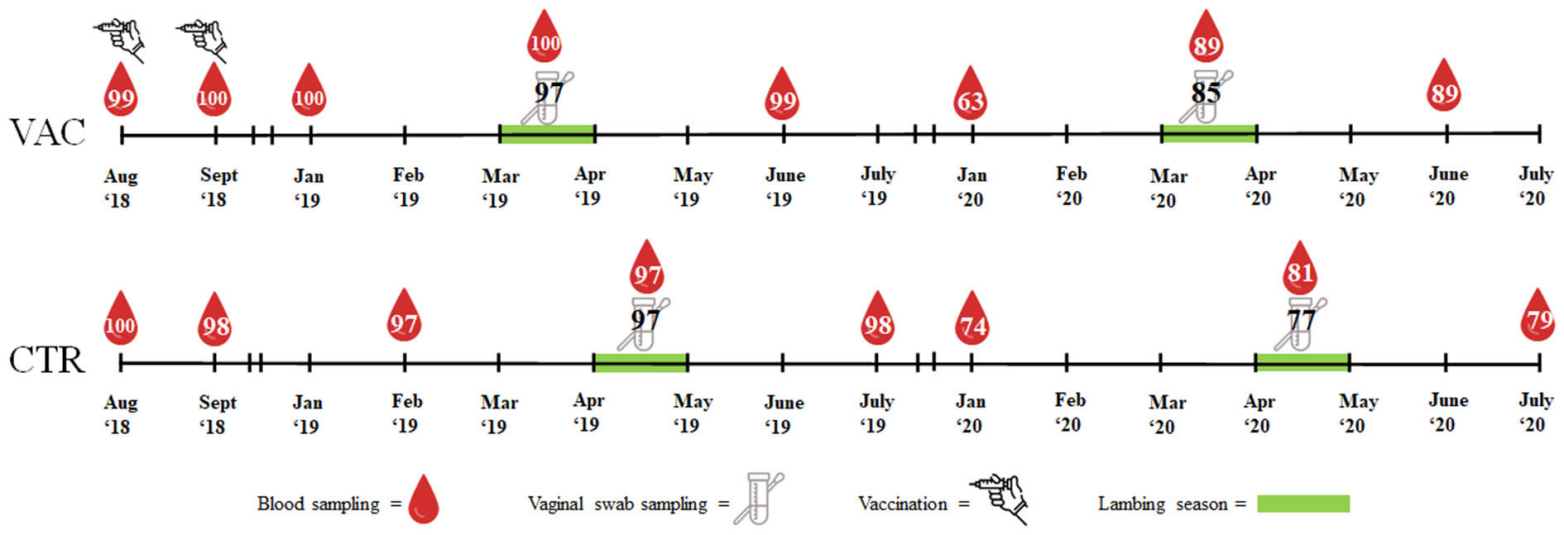
Overview of vaccination, sample types and number of samples in two sheep flocks. VAC, sheep flock vaccinated against *Coxiella burnetii*; CTR, control flock.

In August and September 2018, both study cohorts were blood sampled at the *Vena jugularis* (KABEVETTE^®^, KABE, Nümbrecht-Elsenroth, Germany). Samples were simultaneously collected at the vaccination date of study cohort VAC. Before the lambing seasons started, blood samples were taken from both cohorts in January/February 2019 and in January 2020. During the lambing seasons in 2019 and 2020, blood samples and vaginal swabs (Sarstedt, Nümbrecht, Germany) were taken within three days after the ewes had lambed or aborted. Approximately three months after the lambing season, the sheep were blood sampled again in June 2019/2020 (cohort VAC) and July 2019/2020 (cohort CTR). Blood samples were centrifuged within 6 h of sampling. The vaginal swabs and sera were stored at −18°C until laboratory examination. An overview of the sampling procedure is presented in Figure 1.

### Laboratory examination of serum samples and vaginal swabs

Sheep sera were examined with two phase-specific ELISAs (EUROIMMUN, Lübeck, Germany). Each phase-specific ELISA detected either IgG PhI or IgG PhII antibodies separately. These ELISAs were applied in accordance with the manufacturer's instructions and have been recently described in detail (33). The test results were presented quantitatively in relative Units (RU) determined by a standard curve. The classification was as follows: <16 RU: negative, ≥16 RU till <22 RU: uncertain, and ≥22 RU: positive. This classification applies to both phase-specific ELISA tests.

DNA from the vaginal swabs was extracted with the NucleoSpin Tissue Kit (Macherey Nagel, Düren, Germany) in accordance with the manufacturer's instructions using the KingFisher^TM^ Flex (ThermoFisher Scientific, Dreieich, Germany). *C. burnetii*-specific DNA-fragments were detected by amplifying IS*1111* elements with a real-time PCR (LSI VetMAX^TM^*Coxiella burnetii*, Thermo Fisher Scientific, Germany). The real-time PCR was performed in accordance with the manufacturer, and cycle quantification (Cq) values of ≤ 45 were assessed as positive.

### Reproductive performance

The number of lamb losses due to abortion, stillbirth, dystocia and postnatal death within three days were recorded to evaluate the effect of the *C. burnetii* vaccine on lamb mortality in naturally pre-infected sheep flocks. Live-born lambs were weighed with a baby scale (MTB 200, ADAM, Milton Keynes, UK), and the rectal body temperature of the lamb (mirolife VT 1831, Microlife AG, Widnau, Switzerland) was measured within 6 h after parturition. Simultaneously, a modified Apgar score (48) was applied to assess the vitality of the new born lambs (Table 2). The total scores were assessed as follows: 10 points = excellent condition, 6–9 points = moderately depressed, 0–5 points = severely depressed.

**Table 2:** Modified Apgar scores to assess the clinical status of newborn lambs.

**Score**	**0 Points**	**1 Point**	**2 Points**
Appearance (mouth mucosa)	Blue or pale	Light cyanotic or light pink	Completely Pink
Nursing	Absent	With assistance	By itself
Interdigital reflex (reflex irritability)	Absent	Reduced	Active withdrawal
Activity (muscle tone)	Absent	Weak, lies flat, lifts head	Active motion, standing, lifts head
Respiration	Absent	Slow, irregular breathing	Strong, regular breathing

The percentage of abortion rate, stillbirth rate, lamb mortality, and total lamb losses were determined as previously described by Voigt and colleagues (49), with a slight modification of the total lamb losses including only lambs which had died within three days post-partum. Moreover, the rate of lambs which had died due to dystocia was reported.


$$\begin{array}{l} \text{ Abortion rate in }\%\text{ = }\frac{\text{number of abortion events}}{\text{total number of parturitions }(\text{preterm and at term})}\text{x100 }\\ \text{ Stillbirth rate in }\%\text{ = }\frac{\text{number of stillborn lambs at term}}{\text{total number of lambs born at term}}\text{x100 }\\ \text{ Dystocia rate in }\%\text{ = }\frac{\text{number of lambs dying due to dystocia}}{\text{total number of lambs born at term}}\text{x100 }\\ \text{ Lamb mortality in }\%\text{=}\frac{\text{number of live born lambs dying within 3 days post-partum}}{\text{total number of lambs born alive}}\text{x100 }\\ \text{Total lamb losses in }\%\text{=}\frac{\text{stillborn lambs+lambs dying due to dystocia+number of live born lambs dying within 3 days post-partum}}{\text{total number of lambs born at term}}\text{x100} \end{array}$$

The ewes were also weighed with a livestock scale (WA 200, Meier-Brakenberg, Extertal, Germany) within 6 h after parturition. Three days after lambing, the rectal body temperature (mirolife VT 1831, Microlife AG, Widnau, Switzerland) was taken, and the presence of vaginal discharge was evaluated to receive information about post-partum disorders in the ewes. The extensive husbandry conditions with guard dogs and predators [e.g., ravens (*Corvus corax*)] hampered the assessment of placental expulsion, because placentas were immediately eaten by these animals soon after the sheep's parturition. Therefore, this information was not included in the interpretation of the data due to the small number of observations.

### Necropsy of lambs

During the lambing season in 2019 and 2020, necropsy was performed on aborted, stillborn, and dead lambs from both cohorts by using standardized protocols in accordance with the German Federal Research Institute for Animal Health (50). Samples from the lung, liver, and spleen were taken from each carcass, pooled, and investigated with real-time PCR for the presence of *C. burnetii* as described above for the vaginal swabs. In Germany, *Chl. abortus* is the main abortive pathogen in sheep, and co-infection with *C. burnetii* occurs regularly (23, 49). Therefore, organ pool samples were analyzed for the presence of *Chl. abortus* DNA by real-time PCR in accordance with published protocols (51, 52). Cycle quantification (Cq) values from the *Chl. abortus* PCR of <38 was assessed as positive.

Prior to the start of the lambing season in 2020, an increased number of ewes and yearlings aborted in flock CTR. Therefore, investigations were extended in this flock to identify the abortifacient agent. Abortion material from non-study ewes was included and examined as described above. Moreover, the organ pools were tested for a broader range of pathogens such as *Brucella* sp., *Campylobacter fetus*, Bluetongue Virus, Schmallenberg Virus, and *Neospora caninum*. In addition, brain tissue was tested for the presence of *T. gondii*. In the spring of 2020, Germany was seriously affected by the Covid-19 pandemic, and not all samples could be analyzed for the described pathogens. Details of the performed assays to detect the mentioned abortifacient agents are summarized in Supplementary Table S1.

### Statistical analysis

First of all, data from the serological and molecular analyses as well as the animals' conditions were evaluated descriptively by calculating measures of location scales and dispersion measures.

The IgG PhI and IgG PhII values obtained by the ELISAs from all animals were examined by means of a fixed effects linear model. Herd and sampling time were included in the model and their interaction was added. Since the same animals were sampled at each time point, the sampling time was added in the model as a repeated measure of the ewes examined. Differences between IgG PhI and IgG PhII at each sampling time within each cohort were compared by the Wilcoxon Signed-Rank test. Moreover, the serological results from each seropositive animal at each sampling date were included in further analysis. In detail, the IgG PhII values were divided by the IgG PhI ELISA outcomes. This quotient was established to evaluate the development of phase-specific IgG response against *C. burnetii* during the entire study period of individual sheep. Quotients >1 indicated an increased IgG PhII response, whereas values of <1 demonstrated a higher IgG PhI activity. The McNemar test was used to check for significant differences of the quotient at two consecutive sampling times.

Several Fisher's exact tests were used to check whether there was a significant difference in the number of *C. burnetii* shedding animals in the two flocks and the two lambing seasons in 2019 and 2020.

Data on the condition of the ewes and lambs were examined for significant differences between the cohorts VAC and CTR. An independent two-sample *t*-test was used for continuous variables and a Fisher's exact test was applied for categorical variables.

For all calculations, we used the statistical software SAS (SAS Institute Inc., Cary, NC, USA).

### Ethics approval statement

The procedures on sheep in both flocks were licensed by the federal state governments of Schleswig-Holstein (Az. V 242-39872/2018) and Mecklenburg-Western Pomerania (Az. 7221.3-2-017/18) and were conducted in accordance with German animal welfare legislation and the EU Directive 2010/63/EU for animal experiments. All animals were handled according to high ethical standards and national legislation. Written informed consent was obtained from the owner for the participation of his animals in this study.

Ethical review and approval was not required for the study on human participants in accordance with the local legislation and institutional requirements. Both shepherds provided their written informed consent to participate in this study.

## Results

### Humoral immune response

**Figure 2:**
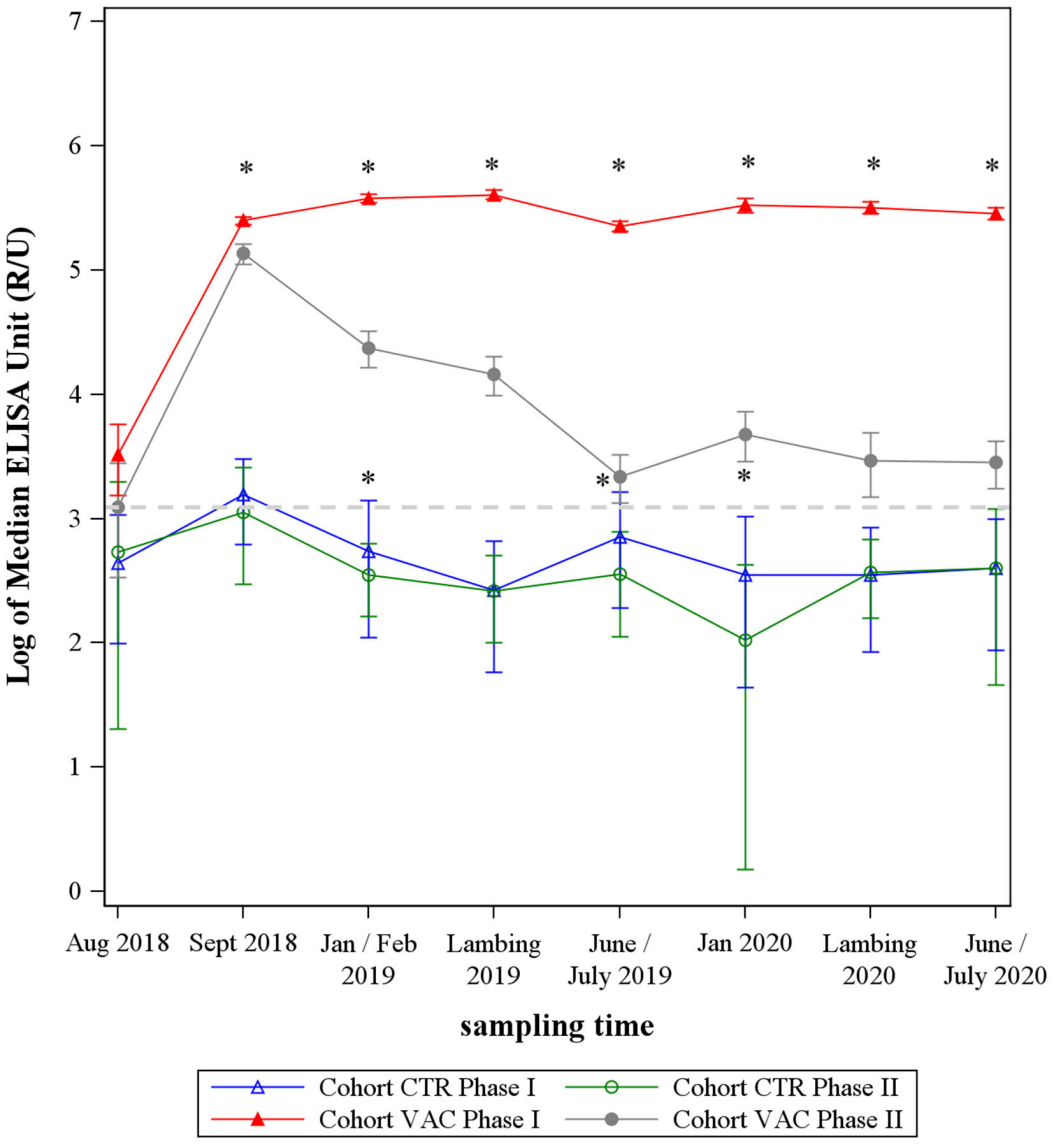
Median levels of *Coxiella burnetii* IgG Phase I and Phase II from two sheep cohorts. VAC: sheep vaccinated against *Coxiella burnetii*; CTR: control sheep; dashed line: cut-off value of both phase-specific ELISAs. *Significant difference between IgG Phase I and IgG Phase II median levels within each cohort (*p*< 0.05).

After vaccinating the cohort VAC, both IgG phase-specific antibodies increased sharply (Figure 2). The IgG PhII response declined continuously after September 2018. The IgG PhI values increased up until the lambing season in 2019 and remained on this high level until the end of the study. In total, median IgG PhI response was always above the median values of IgG PhII after vaccination (*p* < 0.05).

During the entire study period, the median values of cohort CTR remained at a low level and showed an undulating trend, remaining mainly under the ELISAs' cut-off value (Figure 2). The IgG PhI response was significantly higher than the IgG PhII level at three sampling dates (*p* < 0.05) (Figure 2).

On comparing both cohorts, the median levels of *C. burnetii* PhII did not differentiate between the study cohort VAC and CTR at the beginning (August 2018) of the study (*p* > 0.05), but cohort VAC had significantly higher IgG PhI antibodies than cohort CTR in August 2018 (Figure 2). After vaccination and up until the end of the study, the response of both phase-specific IgG's antibodies was significantly higher in cohort VAC compared to CTR (*p* < 0.05).

**Figure 3:**
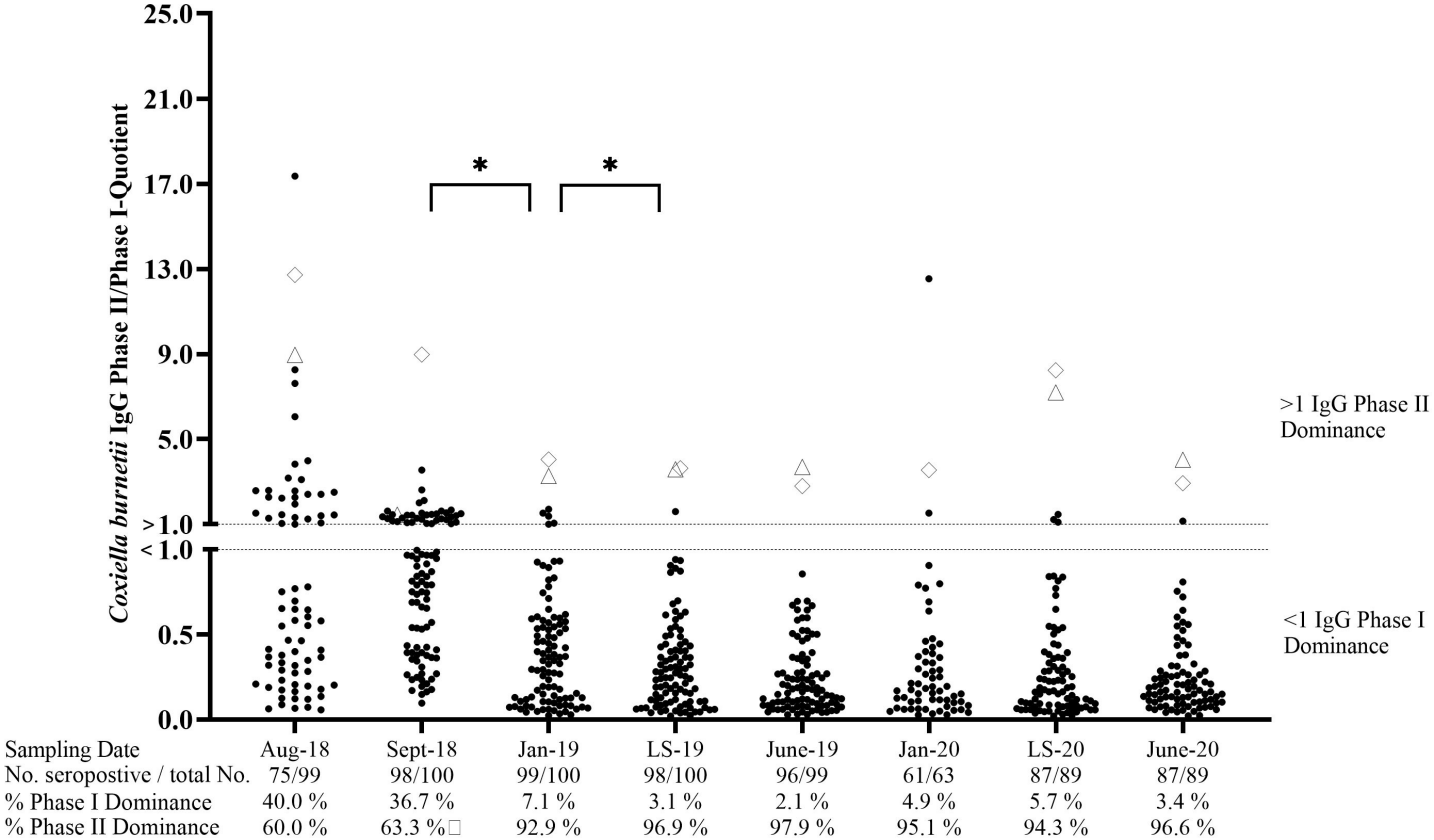
*Coxiella burnetii* IgG Phase II/Phase I quotients of seropositive sheep after vaccination. ♢ and Δ: Two sheep with an IgG Ph II dominance during the entire study period (one missing piece of data in January 2020 for Δ); LS, Lambing season. *Significant difference in phase-specific antibody dominance between two sampling dates (*p*< 0.05).

At the beginning of the study, 75.8% (*n* = 99) of ewes in cohort VAC tested seropositive by at least one of the phase-specific ELISAs (Figure 3). Of these seropositive animals, 40% showed an IgG PhII dominance, whereas 60% had a higher IgG PhI activity. After the first and second vaccinations, the number of sheep with an IgG PhI dominance increased significantly (*p* < 0.05) and remained at a high level (≥94.3%) until June 2020. The IgG PhII levels in two ewes were always higher than the IgG PhI activities during the entire study period (Figure 3). None of the ewes from cohort VAC tested completely negative in the period from August 2018 to June 2020.

**Figure 4:**
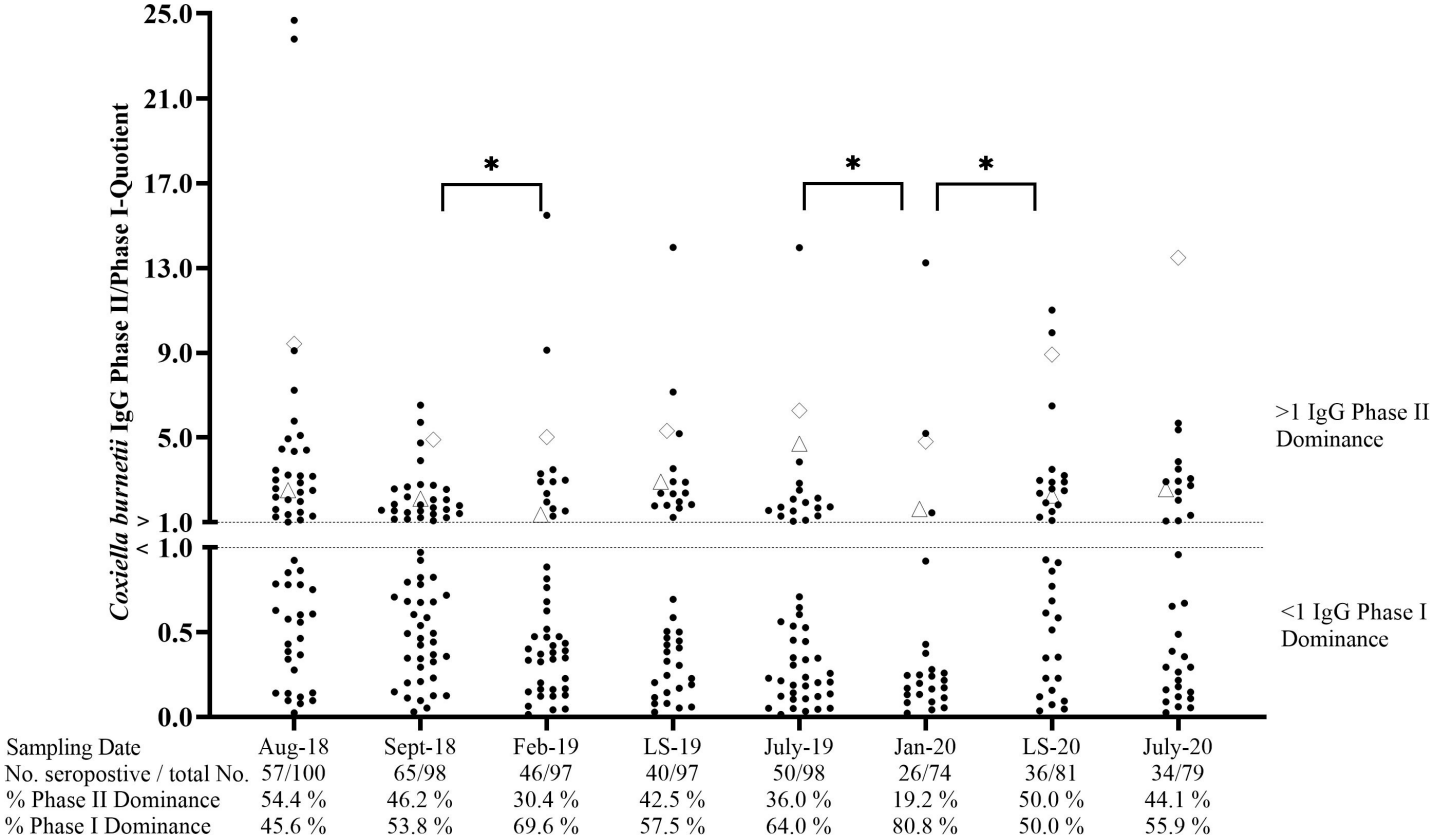
*Coxiella burnetii* IgG Phase II/Phase I quotients of naturally infected sheep. ♢ and Δ: two sheep with an IgG Phase II dominance during the entire study period. LS, Lambing season. *Significant difference in phase-specific antibody dominance between two sampling dates (*p*< 0.05).

Cohort CTR contained 57% seropositive animals in August 2018 (Figure 4). During the following months, the percentage of seropositive ewes showed an undulating development and ranged from 35.1 to 66.3% of ELISA positive animals. The phase-specific investigation also revealed a dynamic pattern with an increasing number of IgG PhI positive sheep from September 2018 to February 2019 and from July 2019 to January 2020. In contrast, the number of animals with an IgG PhII dominance rose during both lambing seasons (LS), but only the surge from January 2020 up until the lambing season in 2020 was significant (*p* < 0.05). In general, the phase-specific response varied in individual animals, but 17 ewes tested completely negative during the entire study period. Moreover, two ewes always had an IgG Ph II dominance during the entire study period (Figure 4).

### Vaginal shedding

The number of vaginal shedders and the detected *C. burnetii* DNA amount on vaginal swabs were low in both cohorts during both lambing seasons. In 2019, significantly fewer sheep shed *C. burnetii* in cohort VAC than in cohort CTR (Table 3). In contrast, there was no significant difference in the number of shedders among VAC and CTR during the lambing season in 2020. None of the sheep excreted the pathogen during either of the lambing seasons. In cohort CTR, five of 12 vaginal shedders had no antibodies against *C. burnetii* at sampling date 2019, and the remaining seven showed an IgG response either to Phase I (*n* = 4), Phase II (*n* = 2), or tested positive with both ELISAs with a higher IgG PhII level (*n* = 1). During the lambing season in 2020, one of the vaginal shedders in the CTR group showed an IgG PhII response, whereas the second positive ewe tested negative with both phase-specific ELISAs. The four vaginal shedders (2019 and 2020) in cohort VAC tested positive with both phase-specific ELISAs post parturition, with an IgG PhI dominance during lambing.

**Table 3:** Vaginal shedding of two *Coxiella burnetii*-infected sheep cohorts.

**Cohort**	**2019**			**2020**		
	**Number of analyzed vaginal swabs**	**Number of *Coxiella burnetii* positive vaginal swabs by PCR**	***p*-value**	**Number of analyzed vaginal swabs**	**Number of *Coxiella burnetii* positive vaginal swabs by PCR**	***p*-value**
CTR	97	12 (Ø 39.8; 34–44)	*p* < 0.0167	77	2 (34,35)	*p* = 1
VAC	97	2 (41,41)		85	2 (36,38)	

VAC, sheep vaccinated against *Coxiella burnetii*; CTR, control sheep; Cq, cycle quantification; numbers in parentheses indicate the Cq values obtained from the vaginal swabs analyzed by real-time PCR; Ø = average Cq value from 12 vaginal swabs with the minimum and maximum result.

### Lamb mortality 2019

In 2019, 98 ewes in cohort CTR and 100 ewes in cohort VAC gave birth to 139 and 148 lambs, respectively. None of the study ewes aborted in 2019. More lamb losses occurred in the vaccinated cohort (*n* = 10) than in the control cohort (*n* = 2). The lamb mortality rate in cohort VAC included four lambs that died due to dystocia, four lambs that were stillborn, two lambs that died within 24 hours post-partum and had previously obtained an Apgar score of 7. Three lambs in cohort VAC (two with dystocia, one post-partum) were necropsied, and none of the organ pool samples tested positive for *C. burnetii*, but *Chl. abortus* (Cq 37) was detected in one lamb (with dystocia). Two lambs in cohort CTR died due to dystocia and were born dead, but further investigations were not performed.

### Lamb mortality 2020

Flock CTR suffered from abortion in 2020 and also five ewes from the study cohort aborted. Among these five ewes, five lambs from three ewes were necropsied and four lambs from two ewes tested positive for *Chl. abortus* (Cq 22, 24, 30, 34). *C. burnetii* was not detected in the five aborted lambs. Two lambs died due to dystocia and were also necropsied and tested negative for *Chl. abortus* and *C. burnetii*. In total, 76 ewes lambed and 107 lambs were born alive, there being no lamb losses within three days post-partum.

Sixteen non-study sheep from flock CTR also aborted and were included in the investigations. Four aborted lambs from four ewes tested *Chl. abortus* positive, and one sheep aborted due to a *T. gondii* infection. *C. burnetii* DNA was not detected from abortion material of these sheep.

Abortion did not occur in cohort VAC during the lambing season in 2020, and this study population had only minor lamb losses (*n* = 4) from a total of 89 lambing ewes. Dystocia (*n* = 3) was the primary cause of lamb losses. Of these four lambs, one lamb was necropsied, but tested negative for *C. burnetii* and *Chl. abortus* DNA. Overall, 122 lambs were born alive in cohort VAC in 2020.

Details about the lamb mortality parameters are presented in Table 4. Moreover, information about the post-mortem results of both lambing seasons are summarized in Supplementary Table S2.

**Table 4:** Lamb mortality parameters during two lambing seasons.

**Lamb mortality parameters**	**CTR 2019**	**VAC 2019**	**CTR 2020**	**VAC 2020**
Total number of parturitions (preterm and at term)	98	100	81	89
Abortion rate (number of abortion events)	0	0	6.2% (5)	0
Total number of lambs at term	140	148	110	126
Stillbirth rate (number of stillborn lambs at term)	0.7% (1)	2.7% (4)	0.9% (1)	0.8% (1)
Dystocia rate (number of dystocia lambs at term)	0.7% (1)	2.7% (4)	1.8% (2)	2.4% (3)
Lamb mortality (number of lambs dying within 3 days pp)	0	1.4% (2)	0	0
Total lamb losses (number of lambs dying at parturition and within 3 days pp)	1.4% (2)	6.8% (10)	2.7% (3)	3.2% (4)

VAC, sheep vaccinated against *Coxiella burnetii*; CTR, control sheep; Cq, cycle quantification; pp, post-partum.

### Conditions of ewes and lambs

The mean body weight of the ewes at parturition did not differ significantly among both cohorts in 2019. In contrast, ewes from cohort CTR were significantly heavier than their counterparts from cohort VAC in 2020 (Table 5). In 2019, the mean body temperature three days after lambing was significantly higher in ewes from cohort CTR than in those from cohort VAC, but there was no significant difference among both cohorts during the subsequent lambing period (Table 5). The number of ewes with vaginal discharge three days post-partum did not significantly differ among the two cohorts during both lambing seasons (Table 5).

**Table 5:** Condition of ewes and their lambs.

	**CTR 2019**	**VAC 2019**	***p*-value**	**CTR 2020**	**VAC 2020**	***p*-value**
**Ewe data**	
Body weight (in kg) 3 days pp mean [95% CI] (*n*)	63.46 [61.92–64.99] (98)	65.51 [63.77–67.26] (99)	*p* = 0.08	63.89 [62.05–65.74] (81)	57.79 [56.21–59.37] (77)	*P* < 0.01
Body temperature (°C) 3 days pp mean [95% CI] (*n*)	39.62 [39.54–39.71] (98)	39.42 [39.32–39.51] (97)	*p* < 0.01	39.29 [39.19–39.40] (81)	39.41 [39.29–39.54] (89)	*p* = 0.16
Vaginal discharge 3 days pp; Yes/No	19/79	21/78	*p* = 0.95	15/66	10/79	*p* = 0.20
**Lamb data**	
Birth weight (in g) live born lambs mean [95% CI] (*n*)	5097.7 [4951.7–5243.7] (138)	4904.8 [4758.5–5051.0] (138)	*p* = 0.06	5107.3 [4927.1–5287.5] (106)	4924.0 [4754.7–5093.3] (122)	*p* = 0.14
Body temperature (°C) in live born lambs mean [95% CI] (*n*)	39.65 [39.58–39.72] (138)	39.77 [39.70–39.84] (135)	*p* < 0.01	39.46 [39.38–39.54] (106)	39.62 [39.52–39.72] (122)	*p* < 0.01

VAC, sheep vaccinated against *Coxiella burnetii*; CTR, control sheep; CI, confidence interval, Cq, cycle quantification; *n*.a., not applicable. Data were not obtained from all sheep.

The mean birth weight of live born lambs did not differ significantly among cohorts CTR and VAC during both lambing seasons. During both lambing periods, lambs from cohort CTR had a significantly lower body temperature post-partum than those from cohort VAC (Table 5).

## Discussion

### Immunization

Vaccination of naturally pre-infected sheep with an inactivated *C. burnetii* Phase I vaccine stimulated the IgG response significantly and IgG PhI levels remained high for two years, whereas IgG PhII response declined after primary vaccination. This is in line with short-term observations from a previous study, and the sharp rise in IgG PhI antibodies after vaccination proves the natural pre-infection of the sheep flock although the proportion of seropositive ewes was very low in April 2018 (33). The long-lasting IgG PhI response raises the question whether booster immunization of vaccinated pre-infected sheep is necessary. Treating mice with purified IgGs from Phase I vaccinated mice was able to inhibit the *C. burnetii* infection and demonstrated the essential contribution of antibodies to the vaccine-induced protection (36). The clearance and complete elimination of the pathogen in the late stage of infection is conducted by T cell-mediated immunity (35, 36). In the present study, we focused on the humoral immune response due to the lacking availability of assays to measure the cell-mediated immune response with a large sample size on a regular basis in veterinary medicine. Nevertheless, immunization with a *C. burnetii* Phase I vaccine stimulated simultaneously both the IgG response and the production of cytokine-producing CD4+ T cells in mice (53). The CD4+ T cells play an important role in signaling B cells and subsequently in triggering an antibody response (37). Therefore, we assume that the ongoing antigenic impact stimulated IgG PhI antibodies and also the cell-mediated response. Consequently, pre-infected sheep do not need a booster vaccination of *C. burnetii* Phase I vaccine after primary vaccination (33). This approach is supported by data recently published by Böttcher and colleagues (34) who controlled Q fever in an infected sheep flock merely due to primary vaccination of the replacement ewes. Future studies are necessary to evaluate the cell-mediated immune response, such as the release of INF-γ after application of a *C. burnetii* Phase I vaccine in sheep in order to confirm our assumptions.

### Serology

Six seronegative non-vaccinated ewes shed the pathogen at parturition, indicating that phase-specific serology is unable to identify vaginal shedders. This is in accordance with results from phase-non-specific ELISAs (46, 54). Moreover, individual ewes from cohorts CTR and VAC showed a dominance of IgG PhII during the entire study period. The lack of seroconversion from high IgG PhII antibodies to an increase in IgG PhI was previously observed in sheep and cattle (33, 55). In cattle, IgG PhII positive animals became seronegative without developing IgG PhI antibodies, but it must be taken into account that the use of different phase-specific ELISA methods hampers a direct comparison (34, 55). In the present study, four sheep (two vaccinated and two non-vaccinated ewes) had high IgG PhII levels with a low IgG PhI response over a two-year period, and these animals did not shed the pathogen at parturition. In humans, high IgG PhII antibody levels also lasted for one year and probably longer after an acute Q fever infection (56, 57). Moreover, higher IgG PhII antibodies were also detected in humans suffering from a persistent *C. burnetii* infection, and pathogen antigen was detected in most cases in bone marrow samples (58). It is suggested that persisting *C. burnetii* antigens cause a continuous immune stimulation (59). Recently, low amounts of *C. burnetii* DNA were detected by qPCR (IS*1111*) in several tissue samples, such as cardiac valves, uterus, and bone marrow from naturally infected sheep (60). Detection of DNA indicates the presence of *C. burnetii* in these organs and might result in constant immune stimulation. However, confirmation of *C. burnetii* organisms in ovine organ samples with advanced diagnostic methods such as fluorescence *in situ* hybridization (61) is still pending and is necessary to support this hypothesis. It would be desirable to measure components of the cell-mediated immunity such as IL-2, IL-10, and IFN-γ, which were previously used to identify a chronic *C. burnetii* infection in mice and humans (62, 63), in order to gain deeper insights into the complexity of persistent *C. burnetii* infection in ruminants. Alongside the constant IgG PhII dominance of the previously described animals, a few ewes showed a similar phase pattern, but for a short period of time, especially in cohort CTR. We cannot rule out that this occurred due to reinfection with *C. burnetii* or serological cross-reactions with other pathogens such as *Chlamydia* spp., *Bartonella* spp., and *Legionella* spp. (64–66). The extensive management system of the sheep flocks allows potential exposure to different pathogens. Hence, evaluation of the disease status based on a single serum sample may lead to misinterpretation, and a second serum sample is needed to confirm the diagnosis as recommended in human medicine (56).

Interestingly, the IgG PhII response in cohort CTR showed an undulating trend with peaks during both lambing seasons. The rise in Phase II antibodies might be associated with high estradiol levels at the end of pregnancy due to the stimulating effect of this hormone on the production of specific antibodies (67). Moreover, it is suggested that estradiol can stimulate antibody production by B cells, probably by inhibiting T cell suppression of B cells (68). The impact of estradiol but also of progesterone on the immune response against *C. burnetii* has already been discussed in goats (30, 69). Therefore, the influence of sexual hormones on pathogen-induced IgG response in small ruminants needs further research.

Taken together, phase-specific serology is a helpful diagnostic tool to characterize the humoral response after naturally acquired *C. burnetii* or vaccination in small ruminants at herd level (31, 33, 34, 70). Nonetheless, it is not able to meet expectations of identifying single shedders and evaluating the disease status of individual sheep.

### Shedding

The *C. burnetii* Phase I vaccine diminished the number of vaginal shedding significantly in the first lambing season, but there was no difference in the second year. Information on the efficiency of the inactivated *C. burnetii* Phase I vaccine in sheep is scarce (71). Astobiza and colleagues (21, 42, 43) conducted studies in *C. burnetii* pre-infected sheep flocks including control groups within the flocks. Leaving non-vaccinated animals in a flock can impact the vaccine efficiency, especially if the antigen is highly contagious, such as *C. burnetii* (42, 72). This might lead to a non-significant reduction in vaginal shedders and the bacterial load among vaccinated and non-vaccinated groups (21, 42). Moreover, self-reduction of *C. burnetii* shedding in sheep flocks has been reported (15, 43, 44), and infected sheep develop a natural immunity against the pathogen. However, gimmers might cause the *C. burnetii* infection to persist in a flock (15). In the present study, the female offspring were not included in the investigations, as we wanted to investigate the effect of the vaccine in multiparous sheep. Furthermore, repeated vaginal shedders were not observed in either sheep flocks, thus indicating that sheep do not develop a persistent *C. burnetii* infection as reported for goats (73).

### Lamb mortality

Only in cohort CTR did ewes abort before the lambing season started in 2020. The abortion rate of 6.2% was higher than reported in sheep flocks from southern Germany and exceeded the critical benchmark of 3%, which is recommended for outbreak investigations (19, 49). Stillbirth and dystocia rates in both flocks were below the median stillbirth rate of 7.2% reported by Voigt and colleagues (49). However, they did not differentiate between stillborn and dystocia. The vaccinated study group had higher total lamb losses (number of losses at parturition and within 3 days post-partum) than the non-vaccinated counterpart. This is mainly associated with the two lambs, which died within 24 hours after lambing. Different farm management systems, sheep breeds, and study designs hamper the comparison of results from other countries. Nevertheless, the lamb losses in our study groups were slight compared to findings from the UK (total lamb losses of 7% and 10%) (74) and Norway (stillbirth rate: ~4%, neonatal mortality ~3%) (75).

During the entire study period, *C. burnetii* was neither diagnosed in aborted fetuses, stillborn lambs or lambs that had died due to dystocia. The main detected pathogen was *Chl. abortus*, which is the main cause of abortion in German sheep flocks (49). Co-infections of *C. burnetii* with other abortive pathogens like *Chl. abortus* and border disease virus were reported, or new infections, e.g., with *T. gondii* can occur during the subsequent lambing season (21, 23, 42). This may lead to possible overestimation of lamb losses due to *C. burnetii* infection in sheep. Therefore, it is essential to analyze abortion material for several pathogens. Moreover, the occurrence of placentitis including the presence of *C. burnetii* organisms in the placenta should be confirmed by microscopical examination in order to confirm a *Coxiella*-associated abortion.

### Conditions of ewes and lambs

The body temperature from ewes in cohort CTR was significantly higher than in cohort VAC during the lambing period in 2019, but the mean values remained within the reference range for adult sheep (39–40.0°C) (76). Although the placental expulsion was not regularly observed because of the extensive management system, the presence of vaginal discharge three days post-partum indicates puerperal disorders such as retention of the fetal membranes (77). The presence of vaginal discharge did not differ significantly among both sheep flocks during the two lambing seasons.

During both lambing periods, lambs from cohort CTR had a significantly lower body temperature than the lambs from the vaccinated flock. A low body temperature at birth is correlated with a low birth weight and this reduces lamb survival (78). However, there was neither a significant difference in birth weight between lambs from both cohorts nor an increased number of postnatal lamb losses in cohort CTR. In general, the mean levels of the lambs' body temperature were within the physiological reference range (39.5–40.5°C) (76).

The authors are aware of the limitations of the investigations. The intra-flock prevalence of *C. burnetii* antibodies, and the amount of *C. burnetii* DNA were low in comparison to other studies (21, 42, 43). Under field conditions, it is challenging to apply a cohort study due to the self-limiting course of Q fever in sheep flocks, and the lack of appropriate diagnostic tools to diagnose *C. burnetii* before shedding. Nevertheless, the low amount of *C. burnetii* shedding was probably sufficient to induce a strong IgG PhII response in shepherd B, and emphasizes the high zoonotic risk for humans to acquire Q fever from infected sheep flocks. Finally, our study investigated the phase-specific IgG response, vaginal shedding, and lamb mortality on a high number of individual sheep over two lambing seasons. With this approach, we gained important insights into the impact of an inactivated *C. burnetii* Phase I vaccine, which plays a key role in disease prevention.

## Conclusions

Taking our findings together, the inactivated *C. burnetii* Phase I vaccine reduces the number of vaginal shedders in a naturally infected sheep flock during the following lambing season and is an efficient control measure. Moreover, immunization stimulates the natural humoral immune response against *C. burnetii* for at least two years in sheep and a booster seems unnecessary. In the future, the cell-mediated immune response has to be evaluated as a primary component of *C. burnetii* defense. For this purpose, novel assays are urgently needed to measure the IFN-γ release on a large scale. Vaccination of infected sheep against *C. burnetii* had no impact on puerperal disorders in ewes and lamb mortality parameters. Therefore, sheep farmers do not have a direct economic benefit from vaccinating their animals against *C. burnetii*. Q fever concerns more public health than the health of sheep. Hence, it is desirable that the public health sector financially supports the vaccination of sheep, in particular of flocks, which are involved in public functions like dyke protection and landscape conservation. This holistic approach is in the spirit of the One Health concept (79). Furthermore, during abortion investigations, it is important to examine aborted fetuses and placentas for several agents, as different pathogens such as *Chl. abortus* or *T. gondii* can co-circulate in a flock and possibly distort the influence of *C. burnetii* on animal health.

## Data availability statement

The original contributions presented in the study are included in the article/Supplementary material, further inquiries can be directed to the corresponding author/s.

## Ethics statement

Ethical review and approval was not required for the study on human participants in accordance with the local legislation and institutional requirements. The patients/participants provided their written informed consent to participate in this study. The animal study was reviewed and approved by the federal state governments of Schleswig-Holstein (Az. V 242-39872/2018) and Mecklenburg-Western Pomerania (Az. 7221.3-2-017/18). Written informed consent was obtained from the owner for the participation of his animals in this study.

## Author contributions

Conceptualization and methodology: BB, MR, and MG. Validation: BB, CS, TH, MR, and MG. Formal analysis, writing—original draft preparation, and visualization: BB and CS. Investigation: BB, CS, TH, SK, and MR. Resources, supervision, project administration, and funding acquisition: MR and MG. Data curation: BB, CS, and TH. Writing—review and editing: TH, SK, MR, and MG. All authors have read and agreed to the published version of the manuscript.
